# A mangrove-based dataset to study the ecology of Neotropical mosquitoes (Diptera, Culicidae) in French Guiana

**DOI:** 10.3897/zookeys.1290.189084

**Published:** 2026-08-25

**Authors:** Médie Collet, Christophe Proisy, Stanislas Talaga, Amandine Guidez, Ioanna Vlandis, Adriana Keovongsack, Romuald Carinci, Pascal Gaborit, Damien Davy, Jean-Bernard Duchemin

**Affiliations:** 1 Unité d’Entomologie Médicale, Vectopôle Amazonien Emile Abonnenc, Institut Pasteur de la Guyane, 23 Avenue Pasteur, 97300, Cayenne, French Guiana, France Unité d’Entomologie Médicale, Vectopôle Amazonien Emile Abonnenc, Institut Pasteur de la Guyane Cayenne French Guiana; 2 AMAP, IRD, Cayenne, French Guiana, France AMAP, IRD Cayenne French Guiana; 3 AMAP, IRD, CIRAD, CNRS, INRAE, Université de Montpellier, Montpellier, France AMAP, IRD, CIRAD, CNRS, INRAE, Université de Montpellier Montpellier France; 4 UAR 3456 LEEISA, CNRS, Université de Guyane, Cayenne 97300, French Guiana, France UAR 3456 LEEISA, CNRS, Université de Guyane Cayenne French Guiana; 5 Arbovirus and Insect Vector Unit, Institut Pasteur Paris, 75015, Paris, France Arbovirus and Insect Vector Unit, Institut Pasteur Paris Paris France

**Keywords:** Biodiversity data, Darwin Core Archive, mosquito assemblages, sampling-event dataset

## Abstract

Understanding how mosquitoes are distributed across natural and anthropogenic environments is crucial but remains challenging in tropical regions. Here, we present a sampling-event dataset designed to document Neotropical mosquitoes (Diptera, Culicidae) assemblages associated with mangrove habitats in French Guiana. A total of 334 collection events were carried out across a variety of coastal and estuarine mangrove habitats that differed in species composition, structure, age, and proximity to urban settlements. The dataset comprises 21,765 mosquito specimens belonging to 14 genera and 62 species. The most abundant taxa were unidentified *Culex* belonging to the subgenus *Melanoconion* (8,580; 39.4%), *Coquillettidia venezuelensis* (4,471; 20.5%), *Deinocerites magnus* (3,354; 15.4%), and *Culex portesi* (1,496; 6.9%), together representing 82.2% of all collected specimens. The dataset includes voucher specimens, associated DNA barcodes, and incorporates male genitalia dissections for selected taxa to support species identifications. This sampling-event dataset provides standardised data on mosquito occurrence and abundance in mangrove ecosystems.

## Introduction

To date, 3,727 valid species of Culicidae have been described worldwide (Harbach 2025). Each species exhibits distinct ecological preferences, ranging from specialisation on a single habitat type to the use of multiple habitats. These ecological differences determine species distributions and habitat associations. As some of these species are important nuisance pests and vectors of pathogens, there is an increasing demand for data on their spatial distribution in habitats located near human populations (Yan and Zhong 2005). The distribution and abundance of these species may influence the epidemiology of mosquito-borne diseases (Wouters et al. 2024), highlighting the need for accurate information on their occurrence and habitat associations. In the Neotropical Region, the number of recorded species is constantly increasing, outpacing our limited knowledge of their ecology. In particular, mangroves, swamps, and tropical rainforests remain largely neglected and undersampled despite harbouring a diverse range of mosquito species (Siwiendrayanti et al. 2020).

French Guiana is recognized as having one of the highest species richness of mosquitos in the world relative to its surface area (Foley et al. 2007). To date, 250 species have been recorded in this overseas territory of France (Talaga et al. 2015a, 2020, 2025; Talaga and Gendrin 2022; Talaga and Duchemin 2023). Georeferenced occurrences of mosquito species in French Guiana were already reported by Fauran and Pajot (Fauran and Institut Pasteur (Cayenne) 1961; Fauran and Pajot 1974) and later updated in 2015 (Talaga et al. 2015b). These databases provide valuable biodiversity and molecular information, but their structure does not support analyses of spatial and temporal variation in species assemblages and relative abundance.

Mangroves are natural and dynamic forest ecosystems that grow in the tropical intertidal zone between 30°N and 30°S (Spalding et al. 2010). These ecosystems also provide habitats for a wide range of mosquito species. Several studies have investigated mosquito occurrence in mangroves, particularly pest and/or arbovirus-carrying species (Ritchie 1992; Knight 2011; Knight et al. 2012; Giordano et al. 2021). On one hand, mangrove habitats represent large water bodies that harbour and support the proliferation of several mosquito species, including pests and arbovirus-carrying species (García-Rejón et al. 2023; Hoyos-López et al. 2016; Talaga et al. 2024). Conversely, they can be poor larval habitats due to frequent tidal and freshwater flooding and flushing, which hinders the oviposition and aquatic developpment of mosquitoes.

In French Guiana, mangrove forests cover approximately 700 km^2^, representing 0.83% of the territory. Nevertheless, they occupy more than 80% of the Guianese coastline, where 90% of the human population lives (Stier et al. 2020). However, information on mosquito species associated with these coastal ecosystems remains limited.

This study presents a standardised sampling-event dataset documenting mosquito assemblages associated with mangrove habitats in French Guiana. The objective was to characterise species occurrence and relative abundance across locations and sampling periods. By combining standardised sampling-event data with specimen-level information, the dataset supports comparisons of mosquito assemblages across space and time while ensuring traceability and open access to biodiversity records through comprehensive specimen and sampling metadata.

## Methods

### Study area

**Figure 1.**
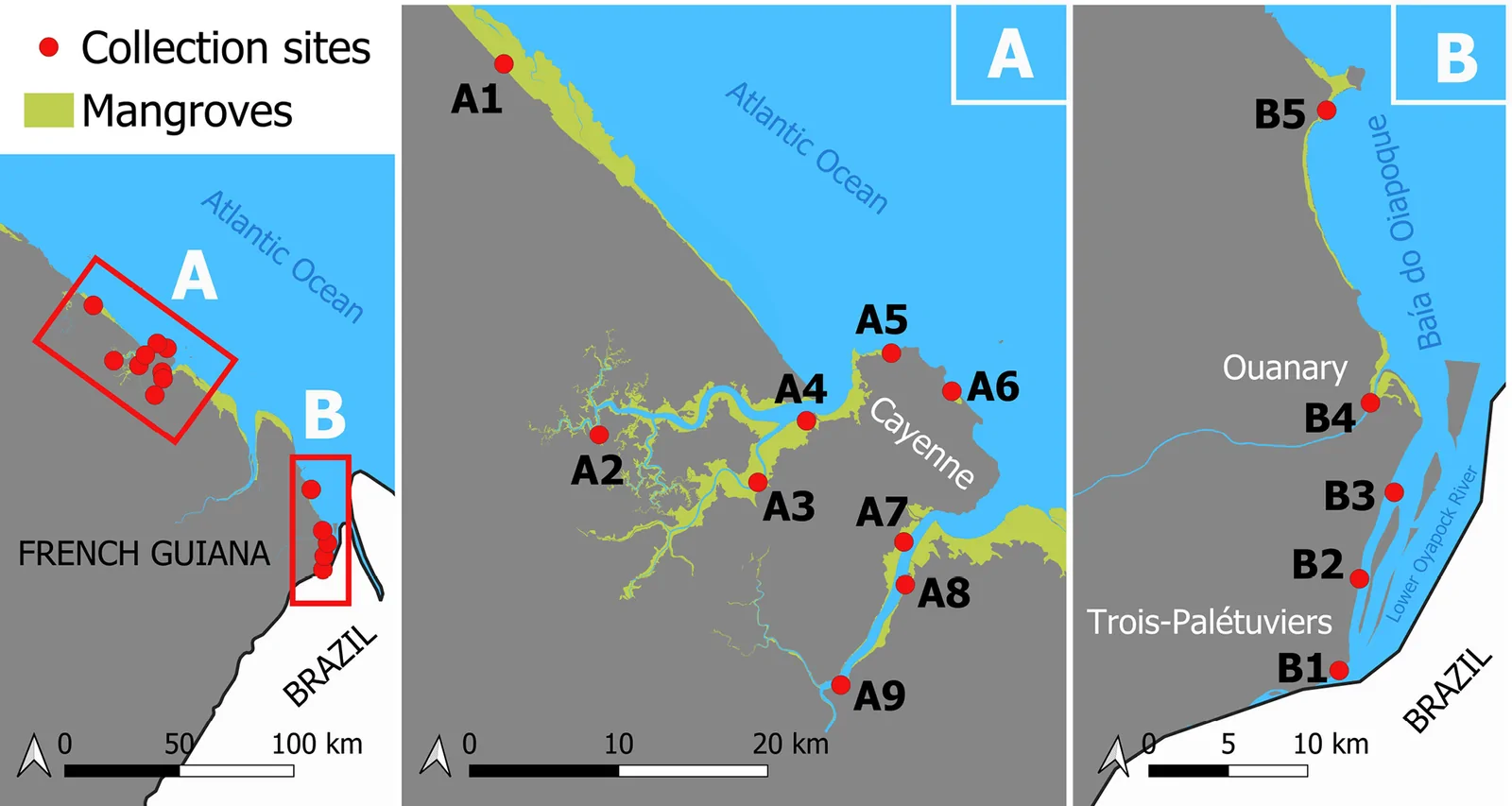
Geographical coverage of mangroves in French Guiana (green areas) and collection sites (red dots) between October 2023 and July 2025. **A**. Sites located on the Island of Cayenne and its direct surroundings; **B**. Sites located along the lower Oyapock River (B). Guatemala (A1); Montsinéry (A2); Petit Cayenne (A3); Larivot (A4); IRD (A5); Salines (A6); La Levée (A7); Auberge du Mahury (A8); Dégrad Roura (A9). Trois-Palétuviers (B1); Berge Gauche (B2); Îlet Perroquet (B3); Dégrad Ouanary (B4); Palétuviers (B5). The scale of the map does not allow for the narrow strips of mangroves to be seen where the three sites on the lower Oyapock are located.

Fourteen locations were sampled in the mangroves of French Guiana (Fig. 1), a French overseas department situated north of Brazil. These locations represented different types of mangrove forest, ranging from young to mature stands, and including both homogeneous and mixed formations with varying structural characteristics.

Most sampling sites were on the Island of Cayenne and its direct surroundings, extending for approximately 50 km along the coast and about 25 km inland. In this area, mangrove forest covers approximately 45 km^2^ within the six municipalities of Kourou, Montsinéry-Tonnégrande, Cayenne, Rémire-Montjoly, Matoury and Roura. A second sampling area was located approximately 100 km southeast of Cayenne, in the lower Oyapock River along the border with Brazil, encompassing a 30 km upstream–downstream transect between Trois-Palétuviers and Ouanary.

The sampled sites differed in their proximity to human settlements, ranging from mangroves directly adjacent to the urban centre of Cayenne and residential or village areas to more isolated sites located over 10 km from human infrastructure. Sampling sites are referenced using codes A1–A9 and B1–B5 (Fig. 1).

**Figure 2.**
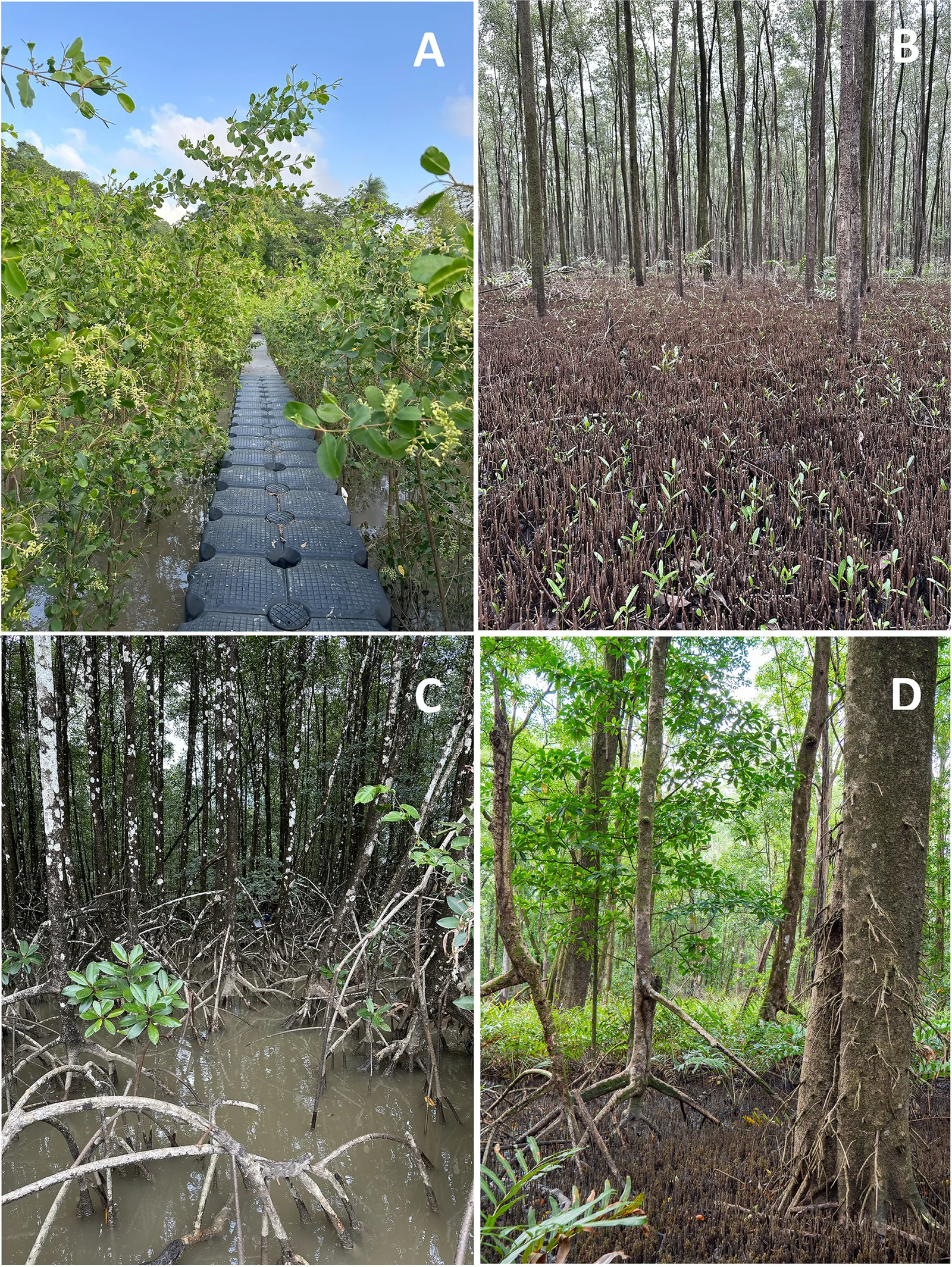
Various mangrove forests sampled on the Island of Cayenne and its direct surroundings. **A**. IRD; **B**. Guatemala; **C**. La Levée; **D**. Petit Cayenne.

At a broader geographical scale, the study region is part of the 1,500 km-long muddiest coastline in the world, in northern South America between the Amazon and the Orinoco River, forming a continuous mangrove-dominated coastal system along the Guianas (Anthony et al. 2011). Along the 320 km of coastline in French Guiana, mangrove forests are dominated by the following tree and shrub species: *Laguncularia racemosa* (L.) C.F. Gaertn., *Conocarpus erectus* L., *Rhizophora mangle* L., *Rhizophora racemosa* G. Mey., and *Avicennia germinans* (L.) L. These species are adapted to saline, anaerobic and highly dynamic environments, and form structurally diverse mangrove stands across the coastline (Fromard et al. 1998; Fromard et al. 2004; Proisy et al. 2007). Mangrove types range from shore-fringed formations, strongly influenced by erosion and sediment accretion processes, to more stable estuarine forests with distinct successional stages (Proisy et al. 2021). The structural and ecological characteristics of the sampled mangrove habitats, including stand age and mangrove tree species composition, are summarised in Table 1 and Fig. 2.

**Table 1.** Features of the sampled mangrove habitats.

**Locality**	**GPS coordinates**	**Features**
A1 (Guatemala)	5°05'48"N, 52°34'09"W	Mature homogeneous mangroves (*Avicennia germinans*) (Fig. 2B)
A3 (Petit Cayenne)	4°51'34"N, 52°23'20"W	Mature heterogeneous mangroves (*Avicennia germinans*, *Rhizophora mangle*) (Fig. 2D)
A4 (Larivot)	4°54'01"N, 52°21'50"W	Mature heterogeneous mangroves (*Avicennia* sp., but *Rhizophora* sp. dominant)
A6 (Salines)	4°55'37"N, 52°16'42"W	Mature homogeneous backshore mangroves (*Rhizophora mangle*)
A5 (IRD)	4°56'46"N, 52°19'02"W	Pioneer heterogeneous mangroves (Fig. 2A), (*Avicennia germinans* and *Laguncularia racemosa*). Established one year before data collection
A2 (Montsinéry)	4°52'43"N, 52°29'15"W	Mature homogeneous mangroves (*Rhizophora mangle*) (Fig. 2C)
A7 (La Levée)	4°49'58"N, 52°17'50"W	
A8 (Auberge du Mahury)	4°48'26"N, 52°17'39"W	
A9 (Dégrad Roura)	4°44'33"N, 52°19'35"W	
B1 (Trois-Palétuviers)	4°03'12"N, 51°39'50"W	
B2 (Berge Gauche)	4°06'20"N, 51°39'32"W	
B3 (Ilet Perroquet)	4°09'25"N, 51°38'43"W	
B4 (Dégrad Ouanary)	4°12'22"N, 51°39'54"W	
B5 (Palétuviers)	4°22'07"N, 51°42'36"W	

At the time of sampling, sites were located between 0 and 1 m above sea level, with an average monthly temperature of 27 °C. The region is characterised by alternating wet and dry seasons. According to Météo-France, four seasonal periods are distinguished: a long rainy season from early April to mid-July, a long dry season from mid-July to mid-November, a short rainy season from mid-November to mid-February, and a short dry season from mid-February to early April.

### Sampling design

Mosquitoes were collected between 23 October 2023 and 11 July 2025. Sampling effort varied according to the objectives of the different sampling campaigns. The dataset comprises three sampling components: (i) a standardised survey conducted in seven mangrove sites on the Island of Cayenne and its direct surroundings, each sampled during four field campaigns using BG-Pro and CDC light traps; (ii) surveys conducted in the Lower Oyapock River, where four sites were sampled along a riverine transect with reduced temporal replication due to logistical constraints; (iii) additional exploratory and opportunistic surveys conducted at several mangrove sites, including preliminary sampling in the Cayenne area prior to the standardised design.

As a result, sampling effort, trap types, operating periods, and replication were not homogeneous across the dataset. Comparative analyses among habitats should therefore be restricted to subsets collected under comparable sampling protocols.

BG-Pro traps baited with BG-Sweetscent and CDC light traps were the main sampling methods (Table 2). Traps were operated during the day (generally for 8–9 h), overnight (generally for 16 h), or occasionally over a continuous 24 h period when field logistics prevented retrieval immediately after the scheduled sampling period. UV light was used in combination with BG-Pro traps during nocturnal sampling, removed during daytime deployments, and occasionally maintained during continuous 24 h trapping session. A summary of sampling effort by collection protocol and mosquito abundance is provided in Suppl. material 1: table SS1. At each site, traps were suspended approximately 1.5 m above ground level and deployed along transects ranging from 30 to 60 m long. Two additional larval dipping events were also conducted and included in the dataset.

**Table 2.** Sampling design, methods, and mosquito abundance in French Guiana mangroves. Additional exploratory samplings included multiple trap types and attractant configurations combining BG-Pro, BG-Sentinel, and CDC light traps with different attractants (BG-Sweetscent, yeast-based lures, dry ice, and UV light), resulting in heterogeneous sampling setups across sites.

**Sampling component**	**Sites (n)**	**Sampling design**	**Traps**	**Events**	**Specimens**
Cayenne standardised sampling	7 sites (A1–A7)	Standardized 4 campaigns/site	BG-Pro (BG-Sweetscent, ± UV)	133	5,372
			CDC light	83	6,956
Lower Oyapock sampling	4 sites (B1–B4)	Structured 2 campaings/site (river–estuary transect)	BG-Pro (BG-Sweetscent UV)	8	1,043
			CDC light	10	3,449
Additional sampling	7 sites (A3, A4, A5, A7, A8, A9)	Exploratory	Multiple trap types and attractant configurations	97	4,921
Larval sampling	(A3, A9, B5)	Opportunistic	Diping	3	64
**Total**				**334**	**21,765**

GPS coordinates were obtained using a Garmin GPSmap 60CSx receiver and the accuracy of the coordinates is estimated to be around 30 m at a 95% confidence level. The geodetic system used was the World Geodetic System 1984 (WGS 84), combined with UTM 21–22 N for map projection.

### Taxonomy and identification

The dataset covers mosquitoes (Diptera, Culicidae). Specimens were identified to species level whenever possible or to the genus level, based on an examination of adult morphological characters. As no single identification key currently covers all mosquito species recorded in French Guiana, species determinations relied on several taxonomic keys listed in Table 3.

**Table 3.** List of mosquito genera ordered alphabetically and used keys for species identification.

**Genus**	**Identification keys**
*Aedeomyia* Theobald	Lane (1953)
*Aedes* Meigen	Home-made key of *Aedes* (08/2007)
*Anopheles* Meigen	Sallum et al. (2020)
*Coquillettidia* Dyar	Home-made key of Mansoniini tribe (02/2007)
*Culex* L.	Lane and Whitman (1951); Berlin and Belkin (1980); Sirivanakarn 1983; Sallum and Forattini 1996
*Deinocerites* Theobald	Adames (1971)
*Haemagogus* Williston	Arnell (1973)
*Limatus* Theobald	Lane (1953)
*Mansonia* Blanchard	Home-made key of Mansoniini tribe (02/2007)
*Psorophora* Robineau-Desvoidy	Home-made key of *Psorophora* (08/2007)
*Sabethes* Robineau-Desvoidy	Neves et al. (2024)
*Toxorhynchites* Theobald	Lane (1953)
*Uranotaenia* Lynch Arribálzaga	Galindo et al. (1954)
*Wyeomyia* Theobald	Lane (1953)

Taxonomic nomenclature and classification follow Talaga et al. 2015a and were updated according to the “Mosquito Taxonomic Inventory” (Harbach 2025). Identifications were carried out using standard entomological procedures applied to adult specimens collected in the field, complemented by genitalia dissection and molecular confirmation for selected specimens.

### Dataset structure and management

Each collection event was assigned a unique identifier (EventID) generated using the abbreviation UEM (Unité d’Entomologie Médicale) followed by a unique collection number. Individual specimen identifiers (OccurrenceID) were derived from the corresponding EventID and completed with a number separated by an en-dash forming a unique specimen code.

All records were assigned to the country “French Guiana” to comply with ISO 3166-1 standards and BOLD recommendations to ensure consistency with international biodiversity databases.

### Processing

Wherever possible, captured specimens were transported alive to the laboratory and killed by freezing at –20 °C. Mosquito specimens were then sorted, identified and stored in 1.5 mL tubes containing 96% ethanol. Alternatively, captured specimens were stored directly in 96% ethanol on site. Fourth-instar larval and pupal exuviae were also sorted and stored in individual tubes containing 70% ethanol. A few adult specimens were mounted on pinpoints attached to No. 3 insect pins by their right side and stored in entomological boxes in the IPG entomological collection.

As most species of the genus *Culex* cannot be reliably identified based on adult external features, 1,762 male *Culex* specimens of the dataset (98% of the total number of *Culex* male specimens) were identified based on the internal structures of the genitalia. To complete this process, the last abdominal segments were cleared in 10% KOH for 2 h at 40 °C, stained in a 1% acid fuchsin solution for 10 min at room temperature, and stored in a solution of Marc André (Cohic and Rageau 1955). In most cases, this process of clearing and staining enabled us to identify *Culex* at species level. In the remaining cases, a total of 100 male genitalia were fully dissected and mounted on microscopic slides in Euparal for examination and identification under a microscope at magnification up to 1000×.

To resolve uncertain species identifications, three legs of the right lateral side of a few specimens were carefully removed and stored in a separate vial containing 96% ethanol and stored at –20 °C for further molecular investigations. This subset comprises 400 female Culicidae identified by DNA barcoding (Hebert et al. 2003) and 50 male *Culex* specimens confirmed using the same approach. After extracting DNA using the DNeasy Blood and Tissue Kit (Qiagen, Valencia, CA, USA), PCR amplification of the standard 658-bp DNA barcode region of the mitochondrial cytochrome c oxidase subunit I (COI) gene was performed using the primers LCO1490/HCO2198 (Folmer et al. 1994). COI sequences were then compared to the DNA reference library dedicated to the mosquitoes of French Guiana (Talaga et al. 2017). Further details regarding the amplification and sequencing protocol used can be found in previous work (Talaga et al. 2017; Talaga and Gendrin 2022). All sequences originating from this dataset will be uploaded to the Barcode of Life Data System (BOLD (Ratnasingham and Hebert 2007)) under the ongoing Mosquitoes of French Guiana (FGMOS) project.

## Results

**Figure 3.**
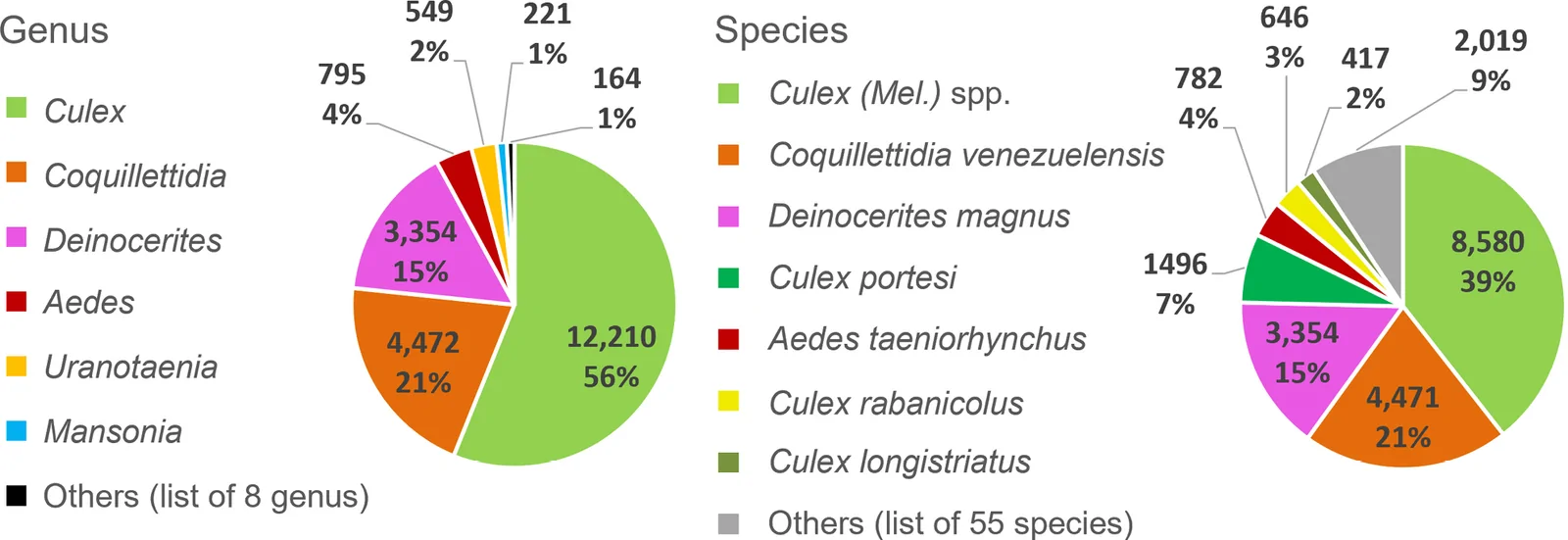
Taxonomic coverage of the dataset by genus (left) and species (right).

A total of 334 sampling events were conducted, resulting in 21,765 mosquito specimens representing 62 species and 14 genera (Fig. 3). The species assemblage was strongly dominated by a few taxa. The most abundant taxa were unidentified *Culex* of the subgenus *Melanoconion* (8,580; 39.4%), *Coquillettidia venezuelensis* (4,471; 20.5%), *Deinocerites magnus* (3,354; 15.4%), and *Culex portesi* (1,496; 6.9%), which together accounted for 82.2% of all collected specimens and occurred in most sampling sites.

Opportunistic collections of immature mosquitoes contributed to a marginal increase of 64 additional specimens. A complete species list with associated abundances is provided in Suppl. material 1: table SS2.

### Advantages and constraints of the sampling plan

Although the overall dataset includes exploratory and opportunistic sampling events, standardised repeated sampling accounted for approximately two-thirds of all collection events, providing a robust basis for comparisons among sites while preserving additional records of rare species.

Diversifying sampling methods by using at least two different types of traps increases the diversity of mosquito species collected. On one hand, CDC light traps are widely used to sample a broad diversity of nocturnal mosquito species (Sudia and Chamberlain 1962; Hoyos-López et al. 2016). On the other hand, BG-Pro or BG-Sentinel traps are effective for sampling anthropophilic mosquito species (Degener et al. 2021). Despite the use of complementary trapping methods, some mosquito taxa are likely to remain underrepresented owing to trap-specific sampling biases.

Immature mosquitoes represent 0.3% of the dataset but were intentionally included. Locating and sampling larvae at the selected sites was time consuming but necessary to identify mosquito larval habitats in mangrove ecosystems.

Environmental conditions vary across mangrove habitats and influence mosquito populations, particularly salt-tolerant species, which are unevenly distributed in space and time (Knight et al. 2012). This dataset was designed to capture mosquito occurrence and abundance across a range of mangrove habitat types, from ground level to canopy.

Human activities occur near mangrove habitats, which are subject to varying degrees of anthropogenic disturbance, distance from urban areas, and conservation status. This variability allows variation in mosquito species occurrence to be assessed across environmental gradients. The dataset provides valuable information on mosquito species occurrence that may support future assessments of public health relevance and management strategies (Knight 2011; Dale and Knight 2012; Brockmeyer Jr et al. 2022).

## Additional information

### Artificial Intelligence (AI) use

The authors accept full responsibility for the content of the manuscript, including the disclosure of any use of AI.

No AI tools were used in the preparation of this manuscript.

### Author ORCIDs

M. Collet https://orcid.org/0009-0008-7669-990X

C. Proisy https://orcid.org/0000-0002-6718-863X

S. Talaga https://orcid.org/0000-0003-1591-4115

A. Guidez https://orcid.org/0000-0003-2821-6447

I. Vlandis https://orcid.org/0009-0008-7915-4147

R. Carinci https://orcid.org/0000-0002-5322-4708

D. Davy https://orcid.org/0000-0002-7054-9643

J.-B. Duchemin https://orcid.org/0000-0001-5044-4855

### Data availability

All of the data that support the findings of this study are available in the main text or Supplementary Information.

## Supplementary materials

Supplementary material 1 Supplementary information Data type docx Explanation note **table SS1**. Summary of sampling effort by collection protocol and mosquito abundance. **table SS2**. List of species and abundance of mosquito specimens collected in mangrove habitats in French Guiana. https://binary.pensoft.net/file/1754203This dataset is made available under the Open Database License (http://opendatacommons.org/licenses/odbl/1.0/). The Open Database License (ODbL) is a license agreement intended to allow users to freely share, modify, and use this Dataset while maintaining this same freedom for others, provided that the original source and author(s) are credited. Médie Collet, Christophe Proisy, Stanislas Talaga, Amandine Guidez, Ioanna Vlandis, Adriana Keovongsack, Romuald Carinci, Pascal Gaborit, Damien Davy, Jean-Bernard Duchemin
