# Supplementary material for: A mangrove-based dataset to study the ecology of Neotropical mosquitoes (Diptera, Culicidae) in French Guiana
Source: Zookeys. 2026 Aug 25;1290:227–39. doi: 10.3897/zookeys.1290.189084 (PMC13535504; doi:10.3897/zookeys.1290.189084)
Supplement: Supplementary file 1 [file zookeys-1290-227_article-189084__-s001.docx]

Supplementary Table S1. Summary of sampling effort by collection protocol and mosquito abundance.

| **Sampling protocol** | **Trap duration (h)** | **Sampling period** | **Day, Night or Day-Night** | **Total nb of collection events** | **Abundance** | **Relative abundance (%)** |
| --- | --- | --- | --- | --- | --- | --- |
| BG-Pro BG-Sweetscent | 8 | 08:00-16:00 | Day | 57 | 60 | 1,0 |
|  | 9 | 08:00-17:00 | Day | 53 | 213 | 9,1 |
| BG-Pro BG-Sweetscent UV | 15 | 17:00-08:00 | Night | 31 | 1981 | 12,3 |
|  | 16 | 16:00-08:00 | Night | 36 | 2682 | 0,3 |
|  | 24 | 16:00-16:00 | Day-Night | 3 | 59 | 2,1 |
|  |  | 08:00-08:00 | Day-Night | 2 | 464 | 6,0 |
|  |  | 09:00-09:00 | Day-Night | 22 | 1302 | 2,6 |
|  |  | 10:00-10:00 | Day-Night | 9 | 565 | 2,0 |
| BG-Sentinel BG-Sweetscent | 16 | 16:00-08:00 | Night | 3 | 431 | 10,1 |
| CDC Light | 15 | 17:00-08:00 | Night | 35 | 2190 | 11,3 |
|  | 16 | 16:00-08:00 | Night | 33 | 2464 | 1,8 |
|  | 22 | 10:00-08:00 | Day-Night | 2 | 402 | 1,2 |
|  | 24 | 08:00-08:00 | Day-Night | 2 | 260 | 10,5 |
|  |  | 09:00-09:00 | Day-Night | 20 | 2277 | 17,6 |
|  |  | 10:00-10:00 | Day-Night | 11 | 3828 | 3,9 |
| CDC Light UV | 16 | 16:00-08:00 | Night | 4 | 853 | 1,1 |
|  | 24 | 16:00-16:00 | Day-Night | 3 | 244 | 0,6 |
| CDC Light UV Dry-Ice | 16 | 16:00-08:00 | Night | 1 | 127 | 4,8 |
| CDC Light UV Yeast | 16 | 16:00-08:00 | Night | 1 | 1055 | 1,1 |
| CDC Light Yeast | 16 | 16:00-08:00 | Night | 1 | 244 | 0,3 |
| Diping | - | - | Day | 5 | 64 | 1,0 |
| **TOTAL** | | | | **334** | **21765** | **100** |

Supplementary Table S2. List of species and abundance of mosquito specimens collected in mangrove habitats in French Guiana.

| **Genus** | **Subgenus** | **Species** | **Abundance** | **Relative abundance (%)** |
| --- | --- | --- | --- | --- |
| *Culex* | *Melanoconion* | spp. | 8580 | 39.4 |
| *Coquillettidia* | *Rhynchotaenia* | *venezuelensis* | 4471 | 20.5 |
| *Deinocerites* |  | *magnus* | 3354 | 15.4 |
| *Culex* | *Melanoconion* | *portesi* | 1496 | 6.9 |
| *Aedes* | *Ochlerotatus* | *taeniorhynchus* | 782 | 3.6 |
| *Culex* | *Melanoconion* | *rabanicolus* | 646 | 3 |
| *Culex* | *Melanoconion* | *longistriatus* | 417 | 2 |
| *Culex* | *Culex* | *declarator* | 224 | 1 |
| *Mansonia* | *Mansonia* | *titillans* | 207 | 1 |
| *Culex* | *Melanoconion* | *idottus* | 173 | 0.8 |
| *Culex* | *Aedinus* | *amazonensis* | 143 | 0.7 |
| *Culex* | *Melanoconion* | *eastor* | 111 | 0.5 |
| *Uranotaenia* | *Uranotaenia* | *lowii* | 110 | 0.5 |
| *Uranotaenia* | *Uranotaenia* | *leucoptera* | 106 | 0.5 |
| *Culex* | *Culex* | *quinquefasciatus* | 100 | 0.5 |
| *Culex* | *Culex* | spp. | 91 | 0.4 |
| *Uranotaenia* | *Uranotaenia* | *geometrica* | 91 | 0.4 |
| *Uranotaenia* | *Uranotaenia* | *apicalis* | 73 | 0.3 |
| *Uranotaenia* | *Uranotaenia* | spp. | 67 | 0.3 |
| *Uranotaenia* | *Uranotaenia* | *nataliae* | 67 | 0.3 |
| *Culex* | *Melanoconion* | *ernsti* | 61 | 0.3 |
| *Aedeomyia* | *Aedeomyia* | *squamipennis* | 37 | 0.2 |
| *Culex* | *Melanoconion* | *phlogistus* | 27 | 0.1 |
| *Culex* | *Melanoconion* | *vomerifer* | 27 | 0.1 |
| *Culex* | *Melanoconion* | *contei* | 23 | 0.1 |
| *Limatus* |  | *durhamii* | 23 | 0.1 |
| *Culex* | *Melanoconion* | *pedroi* | 22 | 0.1 |
| *Uranotaenia* | *Uranotaenia* | *pallidoventer* | 21 | 0.1 |
| *Haemagogus* | *Haemagogus* | *janthinomys* | 19 | 0.1 |
| *Limatus* |  | spp. | 19 | 0.1 |
| *Anopheles* | *Nyssorhynchus* | *aquasalis* | 14 | 0.1 |
| *Culex* | *Melanoconion* | *ocossa* | 14 | 0.1 |
| *Culex* | *Melanoconion* | *albinensis* | 13 | 0.1 |
| *Culex* | *Melanoconion* | *spissipes* | 13 | 0.1 |
| *Mansonia* | *Mansonia* | *pseudotitillans* | 13 | 0.1 |
| *Sabethes* |  | spp. | 12 | 0.1 |
| *Sabethes* | *Sabethoides* | *chloropterus* | 12 | 0.1 |
| *Aedes* | *Stegomyia* | *aegypti* | 8 | <0.1 |
| *Culex* | *Microculex* | *pleuristriatus* | 8 | <0.1 |
| *Culex* | *Melanoconion* | *adamesi* | 6 | <0.1 |
| *Toxorhynchites* | *Lynchiella* | *haemorrhoidalis* | 6 | <0.1 |
| *Uranotaenia* | *Uranotaenia* | *hystera* | 6 | <0.1 |
| *Sabethes* | *Sabethoides* | spp. | 5 | <0.1 |
| *Culex* | *Aedinus* | *accelerans* | 4 | <0.1 |
| *Limatus* |  | *asulleptus* | 4 | <0.1 |
| *Aedes* | *Ochlerotatus* | *serratus* | 3 | <0.1 |
| *Culex* | *Melanoconion* | *flabellifer* | 3 | <0.1 |
| *Psorophora* | *Janthinosoma* | *ferox* | 3 | <0.1 |
| *Uranotaenia* | *Uranotaenia* | *mathesoni* | 3 | <0.1 |
| *Uranotaenia* | *Uranotaenia* | *socialis* | 3 | <0.1 |
| *Wyeomyia* |  | spp. | 3 | <0.1 |
| *Wyeomyia* | *Wyeomyia* | *pertinans* | 3 | <0.1 |
| *Aedes* | *Protomacleaya* | *argyrothorax* | 2 | <0.1 |
| *Culex* | *Culex* | *coronator* | 2 | <0.1 |
| *Culex* | *Culex* | *nigripalpus* | 2 | <0.1 |
| *Haemagogus* | *Haemagogus* | *albomaculatus* | 2 | <0.1 |
| *Uranotaenia* | *Uranotaenia* | *pulcherrima* | 2 | <0.1 |
| *Wyeomyia* | *Dendromyia* | *luteoventralis* | 2 | <0.1 |
| *Coquillettidia* | *Rhynchotaenia* | *albicosta* | 1 | <0.1 |
| *Culex* | *Melanoconion* | *putumayensis* | 1 | <0.1 |
| *Culex* | *Melanoconion* | *erraticus* | 1 | <0.1 |
| *Culex* | *Culex* | *mollis* | 1 | <0.1 |
| *Culex* | *Melanoconion* | *eknomios* | 1 | <0.1 |
| *Mansonia* | *Mansonia* | *humeralis* | 1 | <0.1 |
| **Total** | | | **21765** | **100** |
